# Identification of a Four-Gene Signature Based on Metal Metabolism for Alzheimer’s Disease Diagnosis

**DOI:** 10.3390/genes16111287

**Published:** 2025-10-29

**Authors:** Dandan Huang, Shasha Huang, Yunhan Gao, Linxi Yin, Lijun Pan, Wei Xu

**Affiliations:** School of Advanced Materials Engineering, Jiaxing Nanhu University, Jiaxing 314001, China; 202345895207@jxnhu.edu.cn (D.H.); 202245895108@jxnhu.edu.cn (S.H.); 202345895206@jxnhu.edu.cn (Y.G.); 202245575102@jxnhu.edu.cn (L.Y.); 202345895124@jxnhu.edu.cn (L.P.)

**Keywords:** Alzheimer’s disease, metal metabolism, differentially expressed genes, PPI construction, diagnostic model, biomarker

## Abstract

**Background/Objectives**: Alzheimer’s disease (AD) is a progressive neurodegenerative disorder, and dysregulated metal metabolism in the brain is closely linked to its pathogenesis. **Methods**: In this study, we identified differentially expressed metal metabolism-related genes (DEMGs) associated with AD by integrating data from the GEO dataset GSE132903 and the GeneCards database. Protein–protein interaction (PPI) network analysis was used to identify hub genes, followed by receiver operating characteristic (ROC) curve analysis to assess their diagnostic potential as AD biomarkers. To validate these findings, we performed qRT-PCR experiments on a cellular model and further verified the results using an independent external dataset. Finally, we developed a multigene diagnostic model and constructed a nomogram to predict AD risk. **Results**: The results demonstrated that six out of the ten hub genes achieved an area under the curve (AUC) greater than 0.75, and four genes (*GAD1*, *GFAP*, *SYP*, and *UQCRC2*) showed significant potential as candidate biomarkers for AD after further validation. A multigene diagnostic model based on these genes demonstrated strong predictive performance (AUC = 0.861), and a nomogram with high predictive accuracy (C-index = 0.861) was developed to facilitate individualized AD risk assessment. **Conclusions**: This study identifies four metal metabolism-related genes as promising diagnostic biomarkers for AD and provides a validated multigene model along with a clinically applicable nomogram for individualized risk assessment.

## 1. Introduction

Alzheimer’s disease (AD) is a complex neurodegenerative disorder that primarily affects the elderly population, characterized by cognitive decline and memory loss. The etiology of AD remains multifactorial, and recent research has increasingly focused on the role of metal metabolism in the pathogenesis of AD. Abnormalities in metal homeostasis, particularly involving essential metals such as copper, iron, and zinc, have been implicated in the development and progression of AD [1]. These metals are crucial for various biological processes, including neurotransmitter synthesis, oxidative stress regulation, and cellular signaling. Dysregulation can lead to neurotoxic effects, contributing to neuronal death and cognitive decline [2].

Zinc is an essential trace element that plays a critical role in various biological processes, including neurotransmission and antioxidant defense in the central nervous system (CNS). Studies suggest that zinc deficiency may contribute to neuroinflammation and cognitive decline associated with AD [3]. Epidemiological evidence indicates that zinc supplementation is associated with a reduced risk of developing AD and slower cognitive decline in affected individuals [4]. In animal models, zinc deficiency has been shown to exacerbate memory deficits without altering amyloid-beta (Aβ) plaque burden, suggesting that zinc may influence cognitive function independently of amyloid pathology [5]. Furthermore, zinc interacts with Aβ, promoting its aggregation and potentially contributing to plaque formation [6]. The NLRP3 inflammasome, a key regulator of neuroinflammation, is also modulated by zinc levels, with deficiency enhancing inflammatory responses in microglia [5].

Copper is another essential trace metal that plays a vital role in brain function, particularly in enzymatic reactions and neurotransmitter release. Excess copper can lead to neurotoxicity through the generation of reactive oxygen species (ROS), which contribute to oxidative stress and neuronal damage [7]. Studies have shown that copper can exacerbate zinc-induced neurotoxicity, highlighting the interplay between these two metals in the context of neurodegeneration [8]. The accumulation of copper in the brain has been associated with the formation of amyloid plaques and neurofibrillary tangles, key pathological features of AD [9]. Moreover, copper’s involvement in ferroptosis—a form of regulated cell death driven by iron—further complicates its role in neurodegenerative pathology [10].

In Alzheimer’s disease, iron dysregulation can lead to the generation of hydroxyl radicals via the Fenton reaction, causing oxidative damage to lipids, proteins, and DNA [11]. This oxidative stress is thought to contribute to the pathophysiological processes underlying AD, including neuronal cell death and the formation of amyloid plaques [12]. Studies have demonstrated that iron accumulation correlates with cognitive decline and is implicated in the progression of AD [13]. Furthermore, the presence of ferromagnetic iron in amyloid plaques has been identified, suggesting a potential role in the redox burden associated with neurodegeneration [14].

Recent breakthroughs in bioinformatics have revolutionized AD research by offering innovative approaches and sophisticated analytical tools. The integration of multi-omics data has enabled researchers to comprehensively investigate AD-related biomarkers and elucidate underlying pathological mechanisms. Cutting-edge studies have established that systematic analysis of disease-specific phenotypic characteristics coupled with identification of differentially expressed genes provides an effective framework for discovering novel diagnostic biomarkers [15,16,17,18].

**Figure 1:**
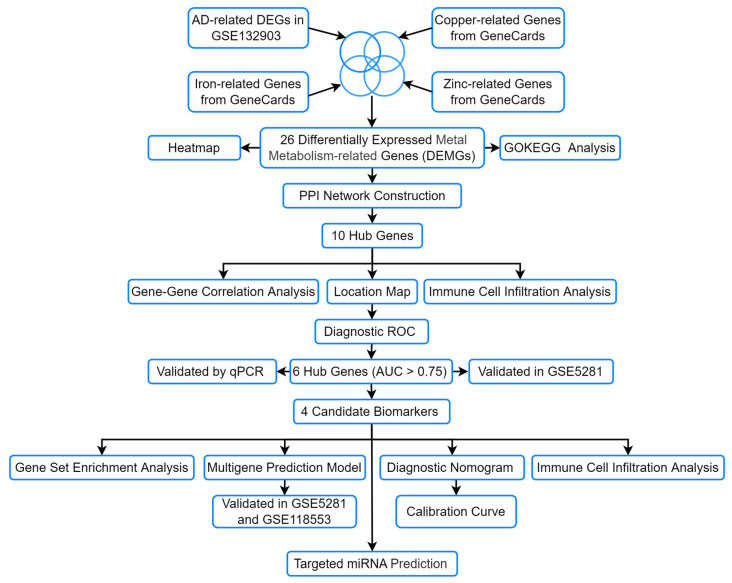
The flow chart of the analyses.

In this study, we identified differentially expressed metal metabolism-related genes (DEMGs) associated with AD by analyzing the GEO dataset GSE132903 and the GeneCards database. Protein–protein interaction (PPI) network analysis was then conducted to pinpoint hub genes, followed by diagnostic receiver operating characteristic (ROC) curve assessment to evaluate their potential as AD biomarkers. To validate these findings, we performed qRT-PCR experiments on a cellular model and further confirmed the results using an independent external dataset. Finally, we developed a multigene diagnostic model and constructed a nomogram to predict AD risk. The overall workflow is illustrated in Figure 1. This study employs a multi-phenotype analytical strategy to identify core genes associated with concurrent dysregulation of copper, iron, and zinc metabolism in AD. The results not only broaden the landscape of potential AD biomarkers but also establish a crucial theoretical framework for future diagnostic advancements and mechanistic exploration of the disease.

## 2. Materials and Methods

### 2.1. Data Acquisition and Preprocessing

Microarray datasets related to Alzheimer’s disease were acquired from the GEO database. The dataset GSE132903 [19] was used to discover biomarkers, and GSE5281 [20,21,22] and GSE118553 [23] dataset were aquired for validation purposes. All the gene expression data were derived from post-mortem brain tissues. Details of the datasets are listed in Table 1. Braak Staging distribution for ADs and controls of GSE132903 was shown in Table S1. Subjects in AD group of GSE5281 dataset had a Braak stage ranging from III to VI. AD participants from GSE118553 are all symptomatic. The detailed experimental protocols for library preparation, hybridization, and RNA quality control are thoroughly described in the original publications for these datasets [19,20,21,22,23]. We removed duplicates and outliers as described in the original literature to determine the final sample for analysis in this study. For the GSE132903 and GSE5281 datasets, we retained all samples for subsequent analysis. For the GSE118553 dataset, we excluded 35 samples from both the AD and control groups based on literature, ultimately using 153 AD samples and 79 control samples for analysis. This study did not include AsymAD samples. Quantile normalization was applied for data standardization using the stats (4.2.1), preprocessCore (1.58.0), and limma (3.54.2) packages in R software (version 4.2.1). The results of data standardization are shown in Figure S1.

Metal metabolism-related gene sets were obtained from the GeneCards database. To ensure high relevance and maintain analytical rigor, only genes with a relevance score > 8 were selected, thereby excluding less significant candidates and refining the dataset for downstream analysis.

### 2.2. Identification of DEGs and DEMGs

Since all data in this study were collected at a single time point, we used a cross-sectional analysis method to assess intergroup differences. Differential expression analysis between AD and control samples was performed using the Limma package in R (4.2.1) [24]. Genes with |logFC| > 0.585 and adjusted *p*-value < 0.05 were considered differentially expressed (DEGs). Volcano plots were generated using ggplot2 (3.4.4) to visualize the DEG distribution. Subsequently, differentially expressed metal metabolism-related genes (DEMGs) were identified by intersecting the DEGs from the GSE132903 dataset with high-confidence DEMGs (GeneCards relevance score > 8). The DEMGs were further illustrated using Venn diagrams and heatmaps. A cross-sectional analysis was performed for our study, as all samples were collected and measured at a single time point.

### 2.3. Biological Functional and Enrichment Analysis of DEMGs

To elucidate the biological significance of DEMGs, we conducted Gene Ontology (GO) and Kyoto Encyclopedia of Genes and Genomes (KEGG) pathway enrichment analyses using the clusterProfiler package (4.4.4) in R (4.2.1) software [25]. The enrichment results were graphically represented using ggplot2 (3.4.4).

### 2.4. PPI Network Construction and Hub Gene Identification

A protein–protein interaction (PPI) network was involved to investigate the potential functional associations among DEMGs. The network was initially generated through the STRING database (https://string-db.org/, accessed on 1 June 2025) [26] with a medium confidence threshold (score > 0.4). Subsequent network analysis and visualization were performed using Cytoscape (3.9.1), where we applied the MCODE analysis module to identify densely connected network clusters under default parameters. Key topological features were analyzed to pinpoint central hub genes within these molecular networks. The association patterns among identified hubs were then statistically evaluated through Spearman correlation analysis, with results graphically represented using the ggplot2 package (3.4.4) in the R software (4.2.1). Predictor importance was quantified using the Random Forest algorithm implemented in the R package randomForest (4.7.7.1). The model consisted of 500 trees, and variable ranking was based on the Mean Decrease in Gini index.

### 2.5. Immune Cell Infiltration Analysis

Immune infiltration analysis was performed using the CIBERSORT algorithm. The gene expression data were input into CIBERSORT with the LM22 signature matrix, which defines 22 immune cell subtypes. The resulting immune cell fractions for each sample were then correlated with the expression levels of target genes by Spearman correlation analysis. A heatmap and lollipop charts were generated to visualize the associations between target genes and immune cell types using the ggplot2 package (3.4.4) in the R software (4.2.1).

### 2.6. Construction of ROC Curves and a Diagnostic Model

To assess the potential of hub genes as diagnostic biomarkers, we employed logistic regression modeling with disease status (AD = 1, control = 0) as the binary outcome variable. The predictive performance was quantitatively evaluated through ROC curve analysis, with diagnostic accuracy determined by calculating AUC values. All statistical computations and graphical representations were implemented using R software (4.2.1), with the pROC (1.18.0) package for analysis and ggplot2 (3.4.4) for plotting. Lasso regression with 10-fold cross-validation was performed to derive coefficients for diagnostic model construction using the glmnet package in R. The multigenic prediction model was constructed in GSE132903, and validated in GSE5281 and GSE118553 datasets. Nomogram model was established using rms (6.4.0), ResourceSelection (0.3–5) in R software. The calibration curve was applied for estimating the accuracy of the nomogram.

### 2.7. Cell Culture and qRT-PCR

The SH-SY5Y neuronal cell line was employed as an established in vitro model and validated by quantitative real-time PCR (qRT-PCR). Cells were maintained in DMEM supplemented with 10% heat-inactivated fetal bovine serum, 100 IU/mL penicillin, and 100 μg/mL streptomycin at 37 °C in a humidified 5% CO_2_ atmosphere. To establish the AD model, cells were treated with 8 μM Aβ1-42 for 12 h. Total RNA was extracted using Trizol Reagent (Takara, Dalian, China), followed by cDNA synthesis with PrimeScript RT Master Mix (Takara, Dalian, China) according to the manufacturer’s protocol.

Quantitative real-time PCR (qRT-PCR) was performed under the following conditions: initial denaturation at 95 °C for 30 s, followed by 40 cycles of 95 °C for 5 s (denaturation), 55 °C for 30 s (annealing), and 72 °C for 30 s (extension) [27]. Relative gene expression levels were quantified using the 2^−ΔΔCT^ method, with GAPDH as the internal reference. Statistical significance was assessed by *t*-test in GraphPad Prism 8.0.0, with significance thresholds set at * *p* < 0.05, ** *p* < 0.01, and *** *p* < 0.001.

### 2.8. External Dataset Validation

The differential expression of qRT-PCR-validated hub genes was further confirmed in the independent dataset GSE5281. Statistical significance was assessed using the Mann–Whitney U test, and results were visualized as violin plots generated with ggplot2 (3.4.4) in R (4.2.1), with additional statistical support from the stats and car (3.1-0) packages.

### 2.9. Gene Set Enrichment Analysis (GSEA)

A single-gene Gene Set Enrichment Analysis (GSEA) was performed to explore the potential signaling pathways associated with four candidate biomarkers. Samples were stratified into high- and low-expression groups according to the median expression level of each hub gene. The analysis was conducted using the “c2.cp.kegg.v2022.1.Hs.symbols.gmt” gene set from the MSigDB database, with gene-level rankings based on log2FC values. Significantly enriched pathways were defined as those with an adjusted *p*-value of less than 0.05. All results were visualized using the ggplot2 package (3.4.4) in R (4.2.1).

### 2.10. Exploration of microRNAs Targeting the Hub Genes

Potential miRNAs targeting the validated hub genes were predicted using the miRDB database (https://mirdb.org, accessed on 1 June 2025). The mRNA-miRNA interaction network was constructed using Cytoscape. miRNAs of high-confidence relevance were screened with a stringent target score threshold (>85), and the association of these candidate miRNAs with Alzheimer’s disease (AD) was further validated using the Human microRNA Disease Database (HMDD) [28].

## 3. Results

### 3.1. Identification of DEMGs

**Figure 2:**
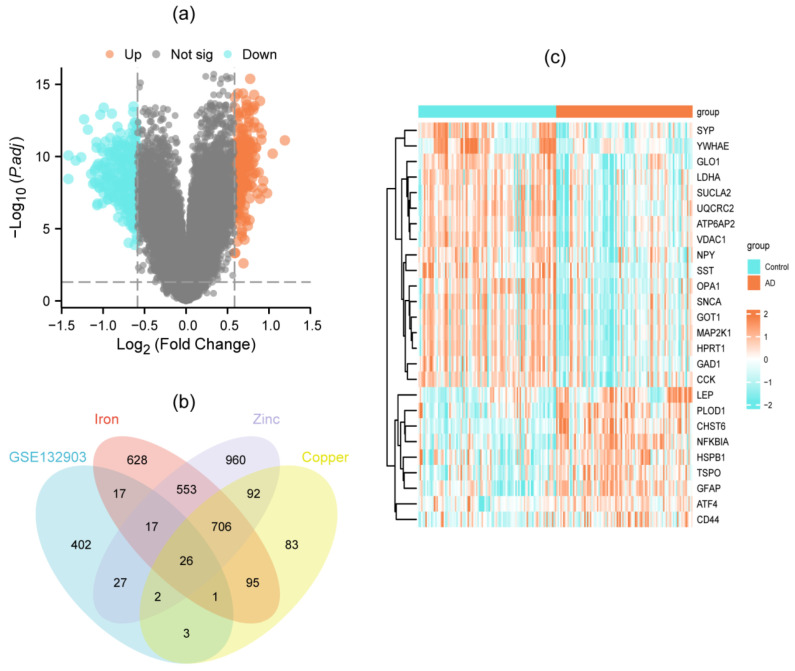
Identification of DEMGs. (**a**) volcano plot exhibiting differential gene analysis in the GSE132903 dataset; (**b**) Venn diagram showing 26 DEMGs; (**c**) heatmap indicating the expression levels of DEMGs in AD and normal samples.

Differential gene expression analysis of the GSE132903 dataset identified 495 DEGs, of which 209 were upregulated and 286 were downregulated. The result was visualized in a volcano plot (Figure 2a). Through stringent screening (relevance score > 8), we obtained metal metabolism-related genes including 2043 iron-, 1008 copper-, and 2383 zinc-associated targets. Intersection with DEGs yielded 26 DEMGs (Figure 2b, Table S2), whose significant differential expression patterns are shown in the heatmap (Figure 2c).

### 3.2. GO and KEGG Enrichment Analysis of DEMGs

**Figure 3:**
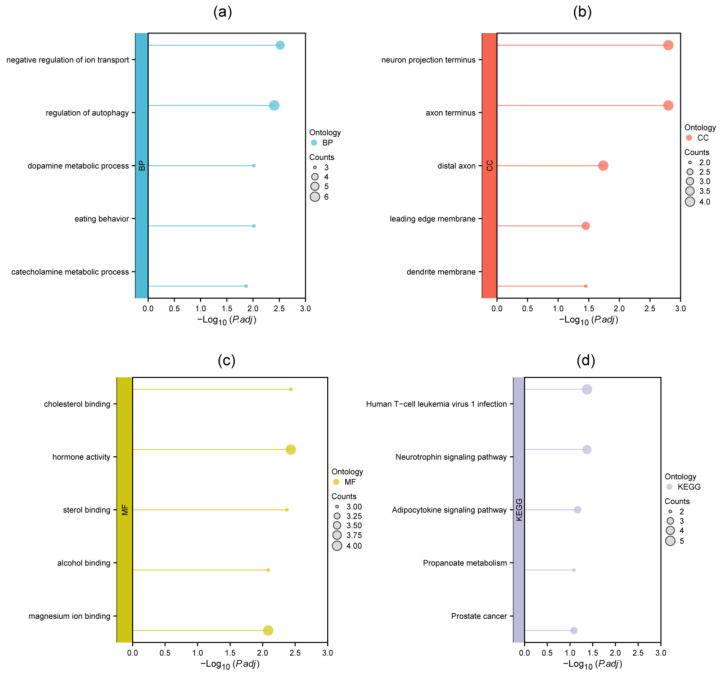
Popsicle chart of GO and KEGG analyses. (**a**–**c**) GO analyses revealed the biological process, cellular component, and molecular function of 26 DEMGs; (**d**) KEGG enrichment analyses indicated involved pathways of 26 DEMGs.

To investigate the potential biological functions of the 26 identified DEMGs, we conducted GO and KEGG enrichment analyses. According to the results, DEMGs were mainly involved in biological process (BP) of negative regulation of ion transport, regulation of autophagy and dopamine metabolic process (Figure 3a). Cellular component (CC) enrichment revealed that DEMGs played a role in neuron projection terminus, axon terminus and distal axon (Figure 3b). Molecular function (MF) mainly comprised cholesterol binding, hormone activity, and sterol binding (Figure 3c). KEGG analysis highlighted key pathways associated with DEMGs, including “Human T-cell leukemia virus 1 infection”, “Neurotrophin signaling pathway” and “Adipocytokine signaling pathway” (Figure 3d). Complete results are documented in Table S3.

### 3.3. PPI Network Construction and Hub Genes Identification

**Figure 4:**
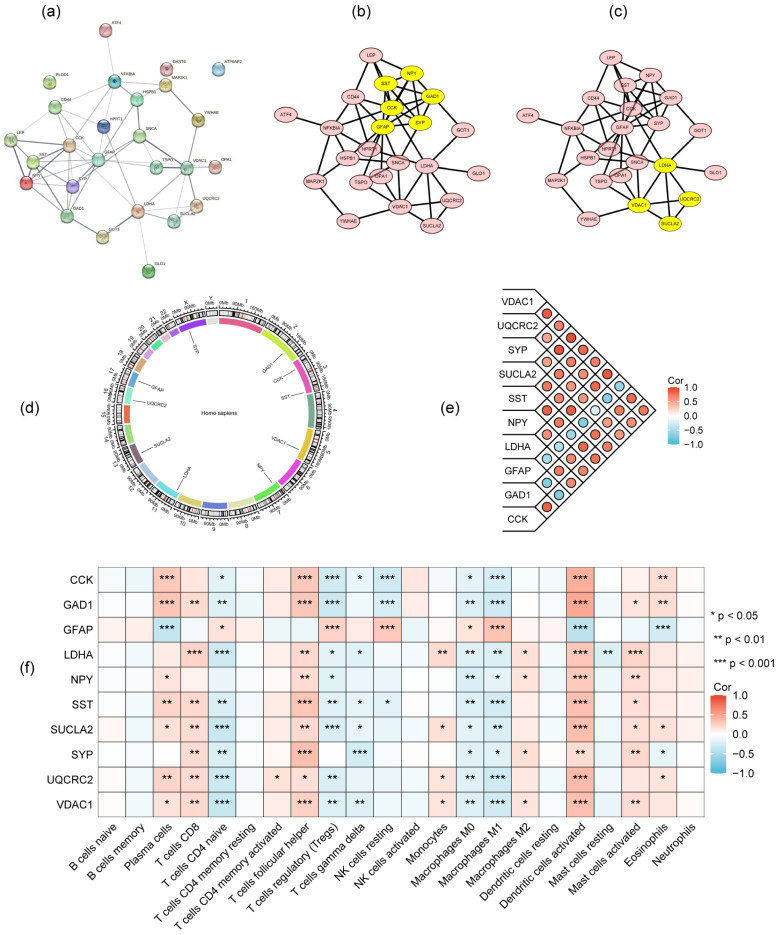
Identification of hub genes. (**a**) PPI network of 26 DEMGs constructed using STRING; (**b**,**c**) 8 hub genes (yellow) in two cluster networks determined using MCODE analysis module of Cytoscape; (**d**) The locations of 10 hub genes on chromosomes; (**e**) The relationships among 10 hub genes revealed by correlation coefficient; (**f**) Immune cell infiltration analysis of 10 hub genes. The colors in (**e**,**f**) represent the correlation coefficients, as indicated in the corresponding captions.

The PPI network of 26 DEMGs was constructed using STRING (Figure 4a) and hub genes were detected using MCODE module after the network data were imported into Cytoscape software (3.10.2). Ten highest-scored genes were identified as the hub genes, which distributed in two cluster networks. *CCK*, *GAD1*, *GFAP*, *NPY* and *SST* were in one cluster, while *LDHA*, *SUCLA2*, *UQCRC2* and *VDAC1* in the other (Figure 4b,c; Table 2). The chromosomal locations of these hub genes are as follows: *CCK* (chr3), *GAD1* (chr2), *GFAP* (chr7), *LDHA* (chr11), *NPY* (chr7), *SST* (chr3), *SUCLA2* (chr13), *SYP* (chrX), *UQCRC2* (chr16), and *VDAC1* (chr5) (Figure 4d). We further investigated the pairwise relationships between the ten hub genes. As the results indicated (Figure 4e, Table 3), *GFAP* showed negative correlations with all nine other genes, with the strongest negative correlation observed between it and *SUCLA2* (correlation coefficient = −0.6582). Conversely, the most significant positive correlation was found between *UQCRC2* and *SUCLA2* (correlation coefficient = 0.9188). Additionally, the correlation heatmap of immune cell infiltration revealed that most of these hub genes were significantly positively correlated with activated dendritic cells, follicular helper T cells, CD8^+^ T cells, activated mast cells and plasma cells, while being significantly negatively correlated with M1 macrophages, M0 macrophages, naive CD4^+^ T cells and regulatory T cells (Tregs) (Figure 4f, Table S4).

### 3.4. Diagnostic ROC Model for Hub Genes

**Figure 5:**
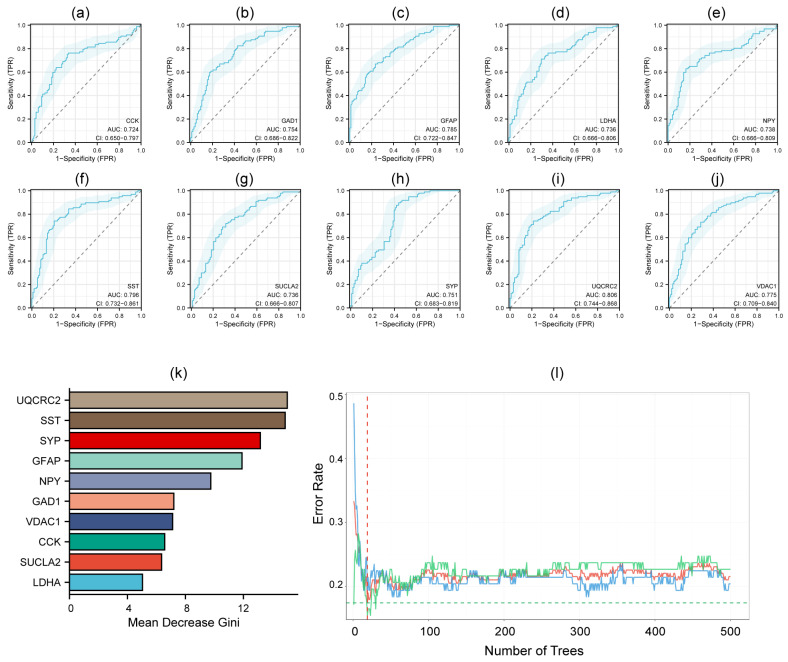
Diagnostic ROC curve of 10 hub genes (**a**–**j**) and random forest ranking results (**k**,**l**). red line: OOB error, blue line: class REF, green line: class TEST.

ROC curve analysis was performed to determine the diagnostic performance of the ten candidate hub genes. The AUC ranges from 0.5 (no discriminative power) to 1.0 (perfect accuracy), where values closer to 1 signify better performance. Analysis revealed that all hub genes achieved AUC scores above 0.7, demonstrating reasonable diagnostic accuracy (Figure 5a–j). Particularly, *UQCRC2* displayed stronger predictive ability with an AUC exceeding 0.8, suggesting high robustness (Figure 5i). Concurrently, we employed Random Forest to rank the importance of these hub genes. An ensemble of 500 decision trees was constructed, and the features were ranked based on the Mean Decrease in Gini index (Figure 5k,l). Based on the random forest ranking results and the diagnostic performance of the 10 hub genes, we ultimately selected six hub genes with AUC values > 0.75 for further cellular experimental verification.

### 3.5. Validation of Hub Genes by qRT-PCR and External Dataset

**Figure 6:**
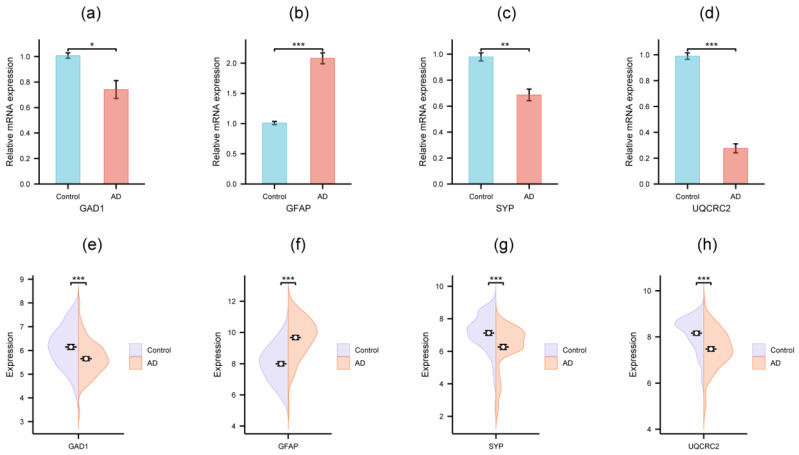
Validation results of four hub genes on SH-SY5Y cell model. (**a**–**d**) Validation by qRT-PCR; (**e**–**h**) Validation in external dataset GSE5281. The black symbols in figures represent the mean ± standard deviation. Significance levels were given as follows: *** *p* < 0.001, ** *p* < 0.01, * *p* < 0.05.

To validate the reliability of our predictions, we selected six hub genes with AUC values > 0.75 for experimental verification in the SH-SY5Y cell model. The primers used in the experiment are listed in Table S5. qRT-PCR analysis revealed that four of these genes exhibited significant differential expression between the AD cell model and the control group (Figure 6a–d). Specifically, *GAD1*, *SYP*, and *UQCRC2* were significantly downregulated in the AD model, whereas *GFAP* was markedly upregulated. To further corroborate these findings, we analyzed the external dataset GSE5281, which confirmed that all four genes showed significant expression differences between AD patients and controls (Figure 6e–h). Notably, the expression trends of *GAD1*, *GFAP*, *SYP*, and *UQCRC2* in both the qRT-PCR experiment and the external dataset aligned with those observed in the GSE132903 dataset (Table 2). These consistent results suggest that these four genes may serve as promising candidate biomarkers for investigating AD mechanisms and aiding clinical diagnosis.

### 3.6. GSEA and Immune Cell Infiltration Analysis of Four Candidate Biomarkers

**Figure 7:**
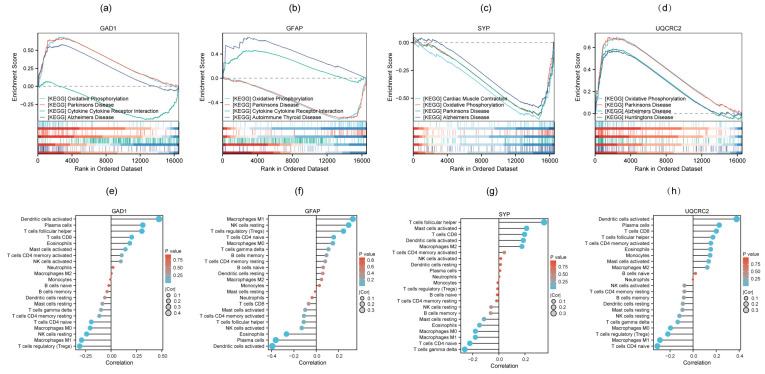
GSEA (**a**–**d**) and Immune cell infiltration analysis (**e**–**h**) of four candidate biomarkers.

Single-gene GSEA results indicated the potential signaling pathways involving the four candidate biomarkers (Table S6), with the top four pathways for each gene displayed in Figure 7a–d. These genes were linked to several neurodegenerative diseases, such as Alzheimer’s disease, Parkinson’s disease and Huntington’s disease. All genes showed enrichment in oxidative phosphorylation, while two of them (*GAD1* and *GFAP*) were closely associated with cytokine-cytokine receptor interaction. To further elucidate the immune infiltration profiles of the 4 candidate biomarkers, a lollipop chart was created to display the extent of infiltration across the 22 immune cell types (Figure 7e–h). *GAD1* showed the strongest positive correlation with activated dendritic cells and the strongest negative correlation with regulatory T cells (Tregs). *GFAP* was most positively correlated with M1 macrophages and most negatively correlated with activated dendritic cells. *SYP* exhibited the strongest positive correlation with follicular helper T cells and the strongest negative correlation with gamma delta T cells. *UQCRC2* was most positively correlated with activated dendritic cells and most negatively correlated with naive CD4^+^ T cells.

### 3.7. Multigenic Prediction Model and Nomogram Construction

**Figure 8:**
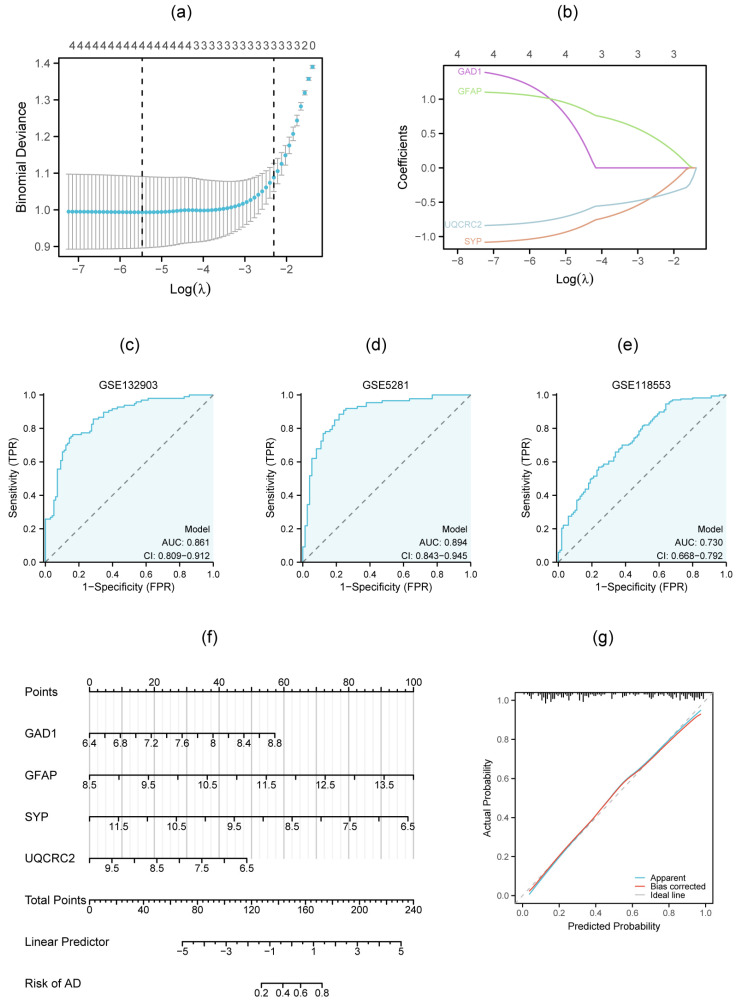
Diagnostic model construction and validation based on four candidate biomarkers. (**a**,**b**) Lasso regression to derive coefficient. purple line: GAD1, green line: GFAP, blue line: UQCRC2, orange line: SYP; (**c**) diagnostic model constructed in GSE132903; (**d**,**e**) diagnostic model validated in GSE5281 and GSE118553. The blue areas represent the area under the curve (AUC); (**f**,**g**) A nomogram model and calibration curve were established for predicting the risk of AD.

Lasso regression was performed to derive coefficients for diagnostic model construction (Figure 8a,b). The diagnostic prediction formula was Y = −3.522 + (1.021 × GAD1) + (1.010 × GFAP) + (−0.995 × SYP) + (−0.7612 × UQCRC2). A multigenic prediction model was constructed based on the four genes in the GSE132903 dataset. The results show that the AUC value of the ROC curves was 0.861, demonstrating the good predictive ability of the model (Figure 8c). Next, we further validated this model in the GSE5281 and GSE118553, and the AUC values were 0.894 and 0.730, respectively, which confirmed the predictive accuracy of this diagnostic model (Figure 8d,e). To further assess AD risk, we constructed a nomogram model integrating the four candidate genes (*GAD1*, *GFAP*, *SYP*, and *UQCRC2*) (Figure 8f). In this model, each gene contributes a specific point value, and the total score obtained by summing these individual values provides a quantitative prediction of AD risk. The model’s predictive accuracy was evaluated using a calibration curve (Figure 8g), which demonstrated excellent discrimination with a concordance index (C-index) of 0.861, indicating robust predictive performance for AD progression.

### 3.8. Identification of miRNAs Targeting Metal Metabolism-Related Biomarkers

**Figure 9:**
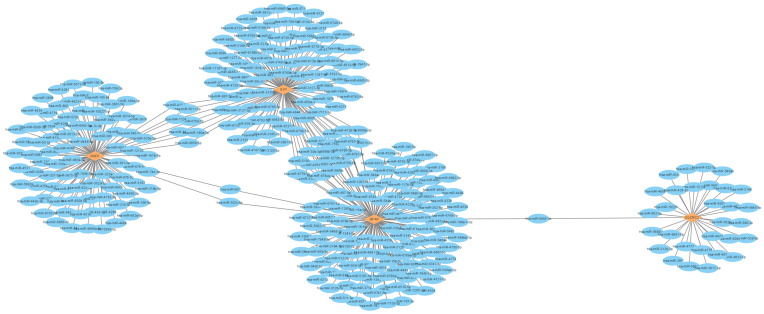
mRNA-miRNA interaction network.

To identify potential miRNA regulators of *GAD1*, *GFAP*, *SYP* and *UQCRC2*, we performed target prediction using the miRDB database. A total of 367 miRNAs were retrieved (Table S7), and the mRNA-miRNA interaction are shown in Figure 9. Subsequently, we selected 71 candidate miRNAs with a stringent cutoff score (>85), and cross-referenced these miRNAs with the HMDD to assess their documented associations with AD. Among them, 10 miRNAs exhibited significant relevance to AD (Table 4), suggesting their potential as therapeutic targets for this disorder.

## 4. Discussion

Alzheimer’s disease is a progressive neurodegenerative disorder and the leading cause of dementia. Growing evidence suggests that AD is closely linked to dysregulated metal metabolism in the brain [40]. Bioinformatics serves as a powerful tool for identifying potential biomarkers associated with AD. In this study, we identified 495 differentially expressed genes (DEGs) by analyzing brain tissue samples from 97 AD patients and 98 healthy controls in the GSE132903 dataset. These DEGs were then intersected with 2043 iron-related, 1008 copper-related, and 2383 zinc-related genes obtained from the GeneCards database, resulting in 26 differentially expressed metal metabolism-related genes (DEMGs). A protein–protein interaction (PPI) network was constructed to identify ten hub genes, the diagnostic potential of which was evaluated using ROC curve analysis. Six genes with an AUC greater than 0.75 were selected for experimental validation via qRT-PCR in a cell model. The results confirmed the differential expression of these genes, among which four (*GAD1*, *GFAP*, *SYP* and *UQCRC2*) showed promise as candidate biomarkers for AD. Furthermore, a multigene diagnostic model demonstrating strong predictive performance (AUC = 0.861) was developed and validated using the GSE5281 and GSE118553 dataset. A nomogram incorporating these four genes was constructed to assess individual AD risk.

This study performed GO and KEGG enrichment analyses on 26 DEMGs, revealing their broad involvement in various biological processes, with the most prominent enrichments observed in energy metabolism and nervous system functions. Copper, an essential trace element, plays a critical role in cellular metabolism, including energy production, antioxidant defense, and neurotransmitter synthesis [41]. Copper deficiency not only leads to mitochondrial dysfunction and impaired ATP synthesis [42], but also disrupts lipid metabolism by reducing fatty acid oxidation capacity [43], thereby aggravating energy metabolism disorders. Iron, indispensable for cellular respiration, is involved in oxygen transport and energy generation. As a key component of cytochromes and iron-sulfur proteins, iron supports efficient electron transfer chain activity and facilitates ATP synthesis. Iron deficiency impairs cellular energy metabolism, compromises mitochondrial function, and diminishes physiological capacity [44]. The GO and KEGG enrichment results further corroborated energy metabolism-related pathways such as adipocytokine signaling, cholesterol binding, and sterol binding, consistent with previous studies. Zinc interacts with multiple neurotransmitter receptors and modulates their activity. For example, zinc binding to glutamate receptors can influence neuronal excitability and play an essential role in synaptic transmission [45]. Zinc is co-released with neurotransmitters and participates in regulating synaptic function and plasticity [46]. Alterations in zinc concentration also affect dopamine synthesis, thereby influencing neural functions related to motor and cognitive processes [47]. Additionally, copper is involved in the synthesis of neurotransmitters such as dopamine and norepinephrine [48]. Functional and pathway enrichment analyses in this study identified terms such as axon terminals, neuron projection terminals, distal axons, dopamine metabolic processes, and neurotrophic signaling pathways, showing strong agreement with established research.

This study identified hub genes with strong diagnostic potential (AUC > 0.75) based on ROC curve analysis, ultimately selecting *GAD1*, *GFAP*, *SYP* and *UQCRC2* as potential diagnostic markers for AD following molecular validation. *GAD1* encodes glutamate decarboxylase 1, an enzyme that catalyzes the decarboxylation of glutamate to produce γ-aminobutyric acid (GABA). As a major inhibitory neurotransmitter, GABA plays a critical role in regulating neuronal excitability and maintaining the balance of neural network activity [49]. Downregulation of GAD1 in AD patients may lead to reduced GABA levels, impairing inhibitory neurotransmission and resulting in neuronal hyperexcitability and cell death [50]. By modulating GABA synthesis, GAD1 not only decreases neuronal excitability but also exerts neuroprotective effects by suppressing excessive neural activity, thereby potentially slowing the progression of AD. Consequently, enhancing GAD1 expression or function could represent a promising therapeutic strategy for AD treatment. *GFAP* encodes glial fibrillary acidic protein, an intermediate filament protein synthesized mainly by astrocytes. Research has shown that elevated GFAP levels are closely associated with cognitive decline in patients with AD, particularly among those diagnosed with mild cognitive impairment (MCI) [51]. Increased GFAP expression is correlated with neuronal damage and cognitive dysfunction, and has been linked to key pathological features such as β-amyloid deposition, tau protein phosphorylation, and neuronal injury. Conversely, suppression of GFAP expression may help alleviate these pathological alterations [52,53]. Studies have further demonstrated that therapeutic strategies targeting GFAP can improve cognitive function in mouse models and reduce levels of AD-related neuroinflammatory markers [54]. *SYP* encodes synaptophysin, a major synaptic vesicle membrane protein involved in vesicle biogenesis and the regulation of neurotransmitter release. The absence of synaptophysin results in a significant reduction in synaptic transmission efficiency [55]. Studies have indicated that decreased levels of synaptophysin are negatively correlated with β-amyloid deposition and the progression of tau pathology, implying that synaptic dysfunction may act as an early biomarker in the pathogenesis of AD [56]. *UQCRC2* encodes ubiquinol-cytochrome c reductase core protein 2, a key subunit of mitochondrial respiratory chain complex III that plays an essential role in electron transport. Deficiency in UQCRC2 leads to mitochondrial dysfunction, resulting in cellular energy deficits and impaired neuronal survival and function-a phenomenon particularly prominent in neurodegenerative disorders such as AD [57]. Moreover, UQCRC2 dysfunction elevates reactive oxygen species (ROS) production, inducing oxidative stress that damages cellular components and promotes apoptotic pathways [58]. Studies have revealed substantial oxidative damage in the brains of AD patients, reflected by increased oxidative modifications of proteins, lipids, and DNA [59]. Therapeutic modulation of UQCRC2 may therefore offer a novel strategy for mitigating pathological states associated with redox imbalance.

Numerous miRNAs have been reported to play important roles in Alzheimer’s disease. In this study, we identified AD-related miRNAs targeting four experimentally validated biomarkers—*GAD1*, *GFAP*, *SYP* and *UQCRC2*—using the miRDB and HMDD. Among the retrieved miRNAs, several have been established as promising AD biomarkers, including hsa-miR-24-3p, hsa-miR-423-5p, and hsa-miR-6734-5p. Others are known to be closely involved in AD-related pathological processes. For instance, hsa-miR-15a-5p was significantly upregulated in the tear fluids of transgenic mice as the disease progressed. Upregulation of miR-195-5p has been linked to tau pathology, while downregulation of hsa-miR-4422, which targets GSAP and BACE1, may promote Aβ plaque formation. Additionally, certain miRNAs, such as hsa-miR-16-5p, exhibit protective effects; overexpression of hsa-miR-16-5p was shown to ameliorate amyloid beta-induced damage by targeting BACE1 in SH-SY5Y cells. Given that these miRNAs also target *GAD1*, *GFAP*, *SYP* and *UQCRC2*, their regulatory mechanisms are likely complex and multifaceted. Further investigation is warranted to explore their potential as therapeutic targets for AD.

The differential expression analysis results in the AD dataset may be associated with factors such as age, sex, and brain cell proportion. After screening out four genes, we incorporated these factors as covariates and constructed four linear models. The results showed that the four genes still exhibited significant differential expression (FDR < 0.05) after controlling for age, sex, and brain cell proportion. Although changes in cell composition explained 40–69% of the epigenetic differences, the remaining effects reflected genuine alterations in gene expression (Figure S2). All genes maintained significant expression across the four models, demonstrating their robustness. The four genes identified in this study show partial correlations with classic AD biomarkers such as *APOE*, *APP*, *MAPT*, *PSEN1*, *PSEN2*, and *BACE1*, neither completely overlapping nor entirely independent. The diagnostic performance of the four-gene combination model (AUC = 0.938) surpasses that of classic biomarkers used alone, indicating that the discovered genes provide incremental diagnostic information (Figure S3). Furthermore, we applied the constructed gene model to predict Parkinson’s disease (PD) in dataset GSE114517, achieving a relatively low prediction accuracy (AUC = 0.613) (Figure S4). Whether this model demonstrates higher specificity for Alzheimer’s disease (AD) requires further investigation in other neurodegenerative disease datasets. These findings further expand and complement the pool of potential AD biomarkers from a distinct perspective, offering additional theoretical support for elucidating the disease mechanisms. There are also some limitations to our study. For instance, the absence of data on medication use and ethnic background in the AD group from the utilized dataset may also serve as potential confounders. In the future, incorporating more datasets and experimental methods will be essential to further validate these results. In addition, future investigations will prioritize the use of explainable AI (XAI) approaches [60] to unravel the specific contribution and interactive logic of these genes within the predictive model.

## Figures and Tables

**Table 1:** The details of three datasets acquired from the GEO database.

Dataset	GSE132903		GSE5281		GSE118553		
Type	Microarray		Microarray		Microarray		
Platform	GPL10558		GPL570		GPL10558		
Genetic source	Middle temporal gyrus		Entorhinal cortex, hippocampus, medial temporal gyrus, posterior cingulate, superior frontal gyrus, primary visual cortex		frontal cortex, temporal cortex, entorhinal cortex, cerebellum		
Groups	Control	AD	Control	AD	Control	AsymAD	AD
Number	98	97	74	87	100	134	167
Age	84.98 ± 6.90	85.02 ± 6.75	79.51 ± 8.92	79.32 ± 7.25	70.44 ± 15.79	86.28 ± 8.59	82.92 ± 10.20
Gender							
Male	50	49	53	51	55	40	69
Female	48	48	21	36	45	94	98

**Table 2:** Ten hub genes selected from DEMGs.

Gene Symbol	logFC	p.adj	Description
*CCK*	−0.61	3.64 × 10^−7^	Cholecystokinin
*GAD1*	−0.99	2.51 × 10^−9^	Glutamate decarboxylase 1
*GFAP*	1.04	6.36 × 10^−11^	Glial fibrillary acidic protein
*LDHA*	−0.61	1.73 × 10^−8^	Lactate dehydrogenase A
*NPY*	−0.65	1.23 × 10^−7^	Neuropeptide Y
*SST*	−0.81	5.91 × 10^−10^	Somatostatin
*SUCLA2*	−0.67	6.31 × 10^−9^	Succinate-CoA ligase ADP-forming subunit beta
*SYP*	−0.95	2.21 × 10^−9^	Synaptophysin
*UQCRC2*	−0.66	7.29 × 10^−12^	Ubiquinol-cytochrome c reductase core protein 2
*VDAC1*	−0.64	1.13 × 10^−9^	Voltage dependent anion channel 1

**Table 3:** Correlation coefficient of 10 hub genes.

Gene Symbol	*CCK*	*GAD1*	*GFAP*	*LDHA*	*NPY*	*SST*	*SUCLA2*	*SYP*	*UQCRC2*	*VDAC1*
*CCK*		0.83	−0.64	0.58	0.67	0.65	0.63	0.61	0.57	0.67
*GAD1*	0.83		−0.66	0.68	0.65	0.77	0.72	0.57	0.70	0.74
*GFAP*	−0.64	−0.66		−0.49	−0.40	−0.50	−0.66	−0.20	−0.65	−0.57
*LDHA*	0.58	0.68	−0.49		0.61	0.63	0.89	0.55	0.85	0.89
*NPY*	0.67	0.65	−0.40	0.61		0.76	0.64	0.57	0.62	0.67
*SST*	0.65	0.77	−0.50	0.63	0.76		0.67	0.67	0.64	0.70
*SUCLA2*	0.63	0.72	−0.66	0.89	0.64	0.67		0.50	0.92	0.92
*SYP*	0.61	0.57	−0.20	0.55	0.57	0.67	0.50		0.48	0.64
*UQCRC2*	0.57	0.70	−0.65	0.85	0.62	0.64	0.92	0.48		0.90
*VDAC1*	0.67	0.74	−0.57	0.89	0.67	0.70	0.92	0.64	0.90	

**Table 4:** Hub genes-targeted miRNAs and the associations between AD.

Gene Symbol	miRNA Name	Description
*GAD1*	hsa-miR-24-3p	Down-regulated in AD [29]; has certain value in the diagnosis of AD and may be a potential biomarker [30].
*GFAP*	hsa-miR-15b-5p	Showed consistent differential expression in AD compared to controls [31].
*GFAP*	hsa-miR-16-5p	Relieved amyloid beta-induced injury by targeting BACE1 in SH-SY5Y cells; protective agents for treatment of Alzheimer’s disease [32].
*GFAP*	hsa-miR-15a-5p	Showed significant up-regulations in the tear fluids of transgenic mice with disease progression, as tracked by cortical Abeta load and reactive astrogliosis [33].
*GFAP*	hsa-miR-195-5p	Upregulation of miR-195-5p might be related to tau pathology [34].
*SYP*	hsa-miR-190a-3p	Significantly upregulated in small neural-derived extracellular vesicles from AD patients [35].
*SYP*	hsa-miR-423-5p	Proposed as new candidate biomarkers in the cross-talk between Diabetes Mellitus and Alzheimer’s Disease [36].
*SYP*	hsa-miR-6734-5p	Remarkably upregulated in the MCI and AD groups; miR-6734-3p and its target mRNA CYTH4 might be used as novel biomarkers for MCI and AD [37].
*UQCRC2*	hsa-miR-4422	Reduced expression of miR4422 that targets GSAP and BACE1 expression can lead to an increase in the formation of Abeta plaque; could be a reliable biomarker for Alzheimer’s diagnosis [38].
*UQCRC2*	hsa-miR-567	Differentially expressed in cerebrospinal fluid samples, blood and serum from MCI-AD patients [39].

## Data Availability

The original contributions presented in the study are included in the article/Supplementary Materials; further inquiries can be directed to the corresponding author.

## Supplementary Materials

The following supporting information can be downloaded at: https://www.mdpi.com/article/10.3390/genes16111287/s1, Figure S1: The results of data standardization; Figure S2: Covariate adjusted analysis; Figure S3: Comparison results with classic AD biomarkers; Figure S4: Four-Gene Signature’s prediction results on the PD dataset (GSE114517); Table S1: Braak Staging distribution for cases (AD) and controls (ND) by sex of GSE132903; Table S2: Information of 26 DEMGs; Table S3: Results of GO and KEGG enrichment analysis for 26 DEMG; Table S4: Results of immune cell infiltraion analysis; Table S5: Primer sequences of mRNA for qPCR; Table S6: Results of GSEA; Table S7: miRNAs from miRDB database targeting the four genes.
