# Supplementary material for: Identification of a Four-Gene Signature Based on Metal Metabolism for Alzheimer’s Disease Diagnosis
Source: Genes (Basel). 2025 Oct 29;16(11):1287. doi: 10.3390/genes16111287 (PMC12652854; doi:10.3390/genes16111287)
Supplement: Supplementary file 1 [file genes-16-01287-s001.zip › Figure S2 Covariate adjusted analysis.pdf]

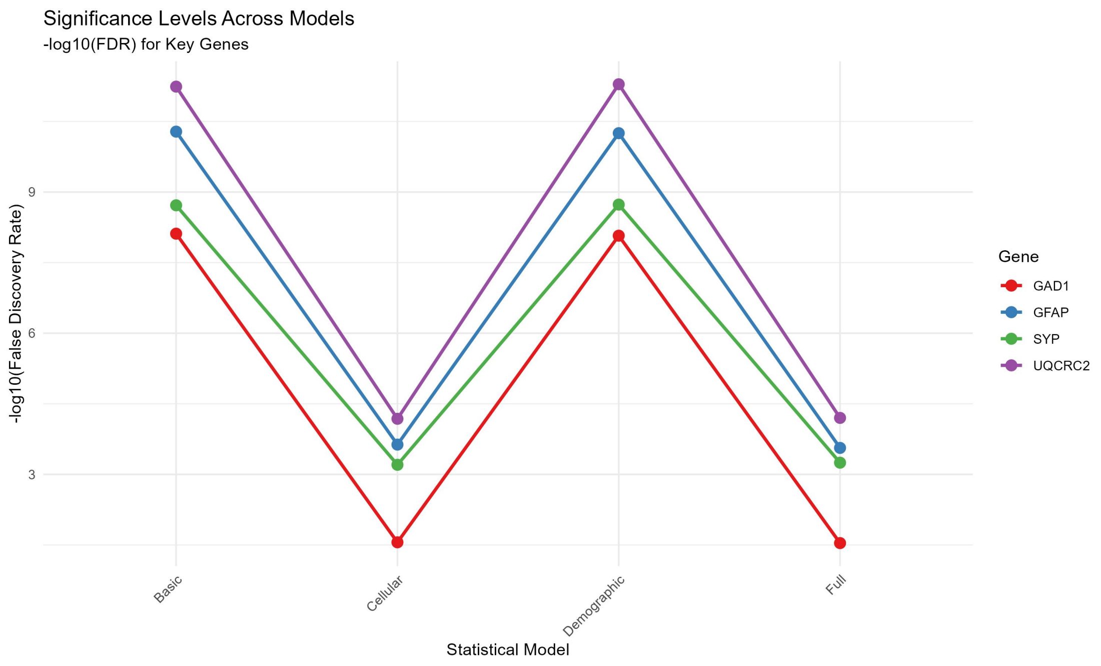

Figure S2 Covariate Adjusted Analysis. The four linear models with covariates are: Basic: Group；Demographic: Group + Age + Gender；Cellular: Group + cell type shifts； Full: Group + Age + Gender + cell type shifts
