# Supplementary material for: Identification of a Four-Gene Signature Based on Metal Metabolism for Alzheimer’s Disease Diagnosis
Source: Genes (Basel). 2025 Oct 29;16(11):1287. doi: 10.3390/genes16111287 (PMC12652854; doi:10.3390/genes16111287)
Supplement: Supplementary file 1 [file genes-16-01287-s001.zip › Figure S3 Comparison results with classic AD biomarkers..pdf]

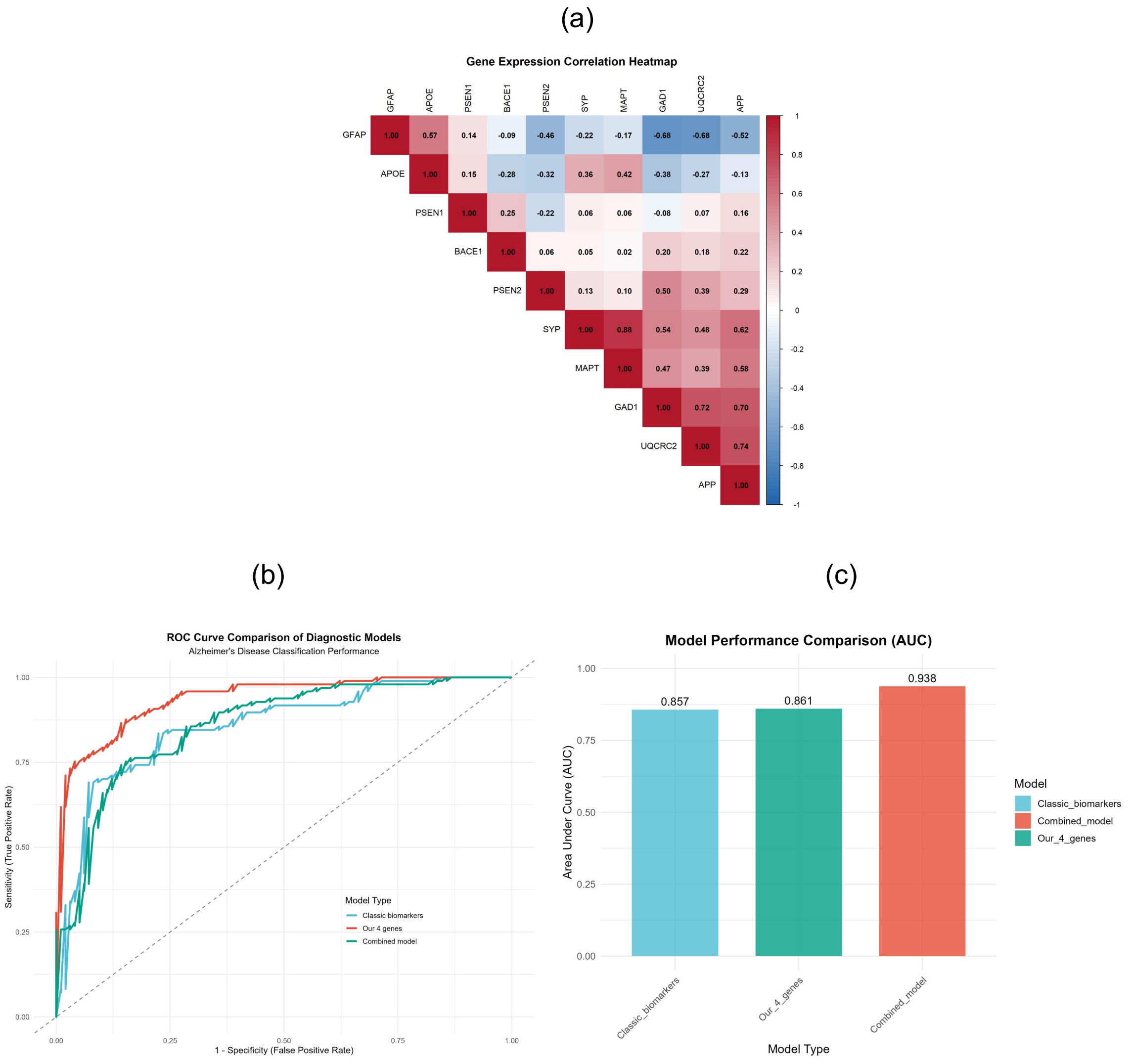

Figure S3 Comparison results with classic AD biomarkers. (a) The correlations between the four genes and classic Alzheimer's disease biomarkers (APOE, APP, MAPT, PSEN1, PSEN2, and BACE1). (b) ROC curve comparison of three diagnostic models (classic biomarkers, our four genes, and combined model). (c) The results of model performance comparison (AUC) among three diagnostic models.
