# Supplementary material for: Identification of a Four-Gene Signature Based on Metal Metabolism for Alzheimer’s Disease Diagnosis
Source: Genes (Basel). 2025 Oct 29;16(11):1287. doi: 10.3390/genes16111287 (PMC12652854; doi:10.3390/genes16111287)
Supplement: Supplementary file 1 [file genes-16-01287-s001.zip › Figure S4 Four-Gene Signature's prediction results on the PD dataset (GSE114517) .pdf]

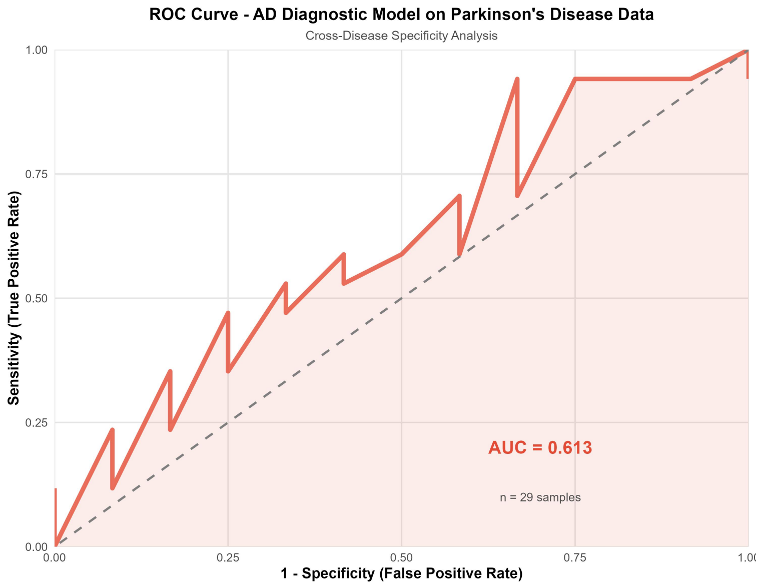

Figure S4 Four-Gene Signature's prediction results on the PD dataset (GSE114517). A total of 29 samples were collected from the Substantia Nigra (SN) region, with a PD: Control ratio of 17:12.
