# Supplementary material for: Identification of a Four-Gene Signature Based on Metal Metabolism for Alzheimer’s Disease Diagnosis
Source: Genes (Basel). 2025 Oct 29;16(11):1287. doi: 10.3390/genes16111287 (PMC12652854; doi:10.3390/genes16111287)
Supplement: Supplementary file 1 [file genes-16-01287-s001.zip › Table S5 Primer sequences of mRNA for qPCR.pdf]

Table S5. Primer sequences of mRNA for qPCR

**Gene symbol**	**Primer sequence**	
	**Forward（5’->3’）**	**Reverse （5’->3’）**
GAPDH	CCAGCCCAGCAAGGATACTG	GGTATTCGAGAGAAGGGAGGGC
GAD1	GCAGAGCCGAGCCTGTTTC	TAGTGGTATTGGGGTCCGCT
GFAP	CTGAGCTGCGAGAGCTGC	TTCATCCTGGAGCTTCTGCC
SST	CGGGGAAGCAGGAACTGG	CTCTGCAGCTCAAGCCTCAT
SYP	GAAGGTGCTGCAATGGGTCT	GCCTGAAGGGGTACTCGAAC
UQCRC2	CCTGCGGGGTGATGTTGATA	CTGCGGATTCTGAAAGGCCA
VDAC1	CCCGGAAGGCAGAAGATGG	GCTTTATTAAGCCAAATCCATAGCC
