# Supplementary figures and images for: Identification of a Four-Gene Signature Based on Metal Metabolism for Alzheimer’s Disease Diagnosis
Source: Genes (Basel). 2025 Oct 29;16(11):1287. doi: 10.3390/genes16111287 (PMC12652854; doi:10.3390/genes16111287)

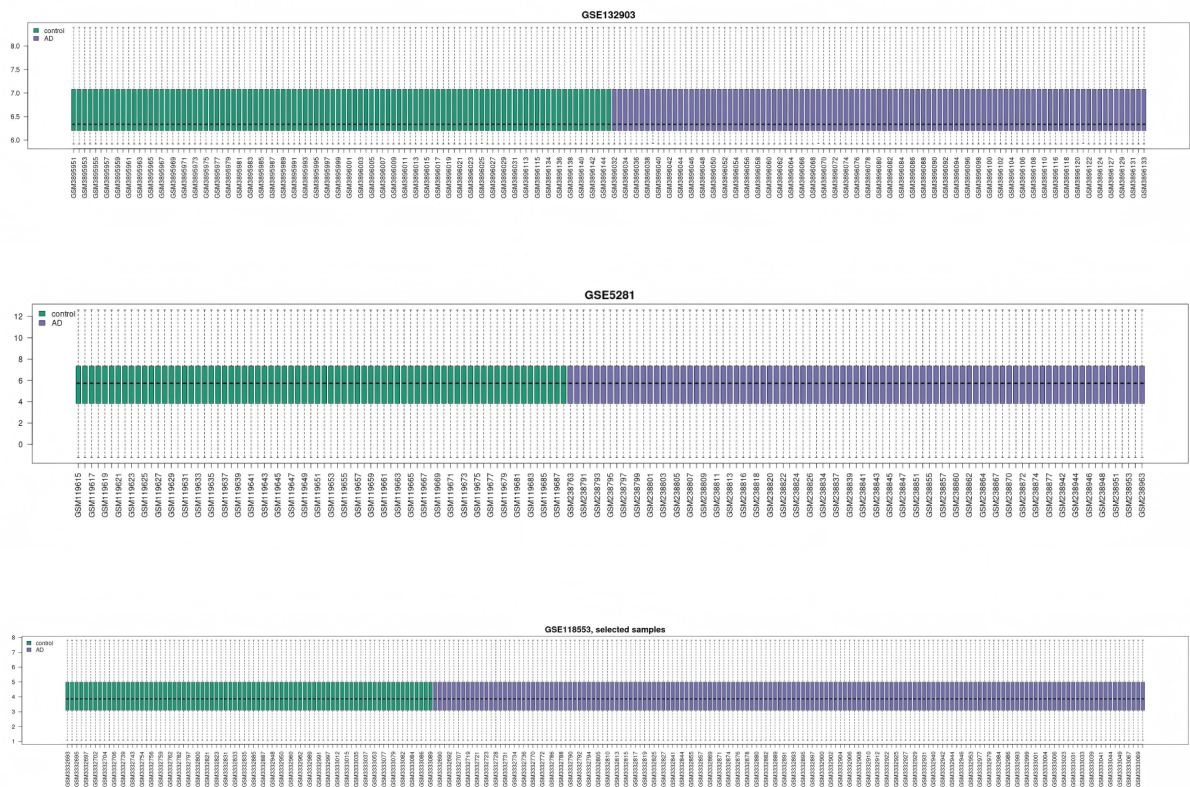

Figure S1 The results of data standardization

Supplement: Supplementary file 1 [file genes-16-01287-s001.zip › Figure S1 The results of data standardization.pdf]
